# Can Magnesium Enhance Exercise Performance?

**DOI:** 10.3390/nu9090946

**Published:** 2017-08-28

**Authors:** Yijia Zhang, Pengcheng Xun, Ru Wang, Lijuan Mao, Ka He

**Affiliations:** 1 Department of Epidemiology and Biostatistics, School of Public Health—Bloomington, Indiana University, Bloomington IN 47405, USA; yz201@iu.edu (Y.Z.); pxun@indiana.edu (P.X.); 2 Key Laboratory of Exercise and Health Science of Ministry of Education, Shanghai University of Sport, Shanghai 200438, China; maolijuan@sus.edu.cn; 3 School of Physical Education and Training, Shanghai University of Sport, Shanghai 200438, China

**Keywords:** magnesium, diet, supplement, exercise performance

## Abstract

Magnesium (Mg) is an essential mineral that plays a critical role in the human body. It takes part in the process of energy metabolism and assists the maintenance of normal muscle function. A number of studies evaluated the association between Mg status/supplementation and exercise performance and found that the need for Mg increased as individuals’ physical activity level went up. Animal studies indicated that Mg might improve exercise performance via enhancing glucose availability in the brain, muscle and blood; and reducing/delaying lactate accumulation in the muscle. The majority of human studies focused on physiological effects in blood pressure, heart rate and maximal oxygen uptake (VO_2_ max), rather than direct functional performances. Some cross-sectional surveys demonstrated a positive association between Mg status and muscle performance, including grip strength, lower-leg power, knee extension torque, ankle extension strength, maximal isometric trunk flexion, rotation, and jumping performance. Additionally, findings from intervention studies showed that Mg supplementation might lead to improvements in functional indices such as quadriceps torque. Moreover, Mg supplementation could improve gait speed and chair stand time in elderly women. This comprehensive review summarized the literature from both animal and human studies and aimed to evaluate scientific evidence on Mg status/supplementation in relation to exercise performance.

## 1. Introduction

Magnesium (Mg) is an essential mineral that plays a critical role in the human body [1]. It is a cofactor for numerous enzymatic reactions including energy metabolism [2], cell growth [3], glycolysis [4], and protein synthesis [5]. Mg is present as the Mg^2+^ ion, which can bind with adenosine triphosphate (ATP) to form the Mg-ATP complex [6]. The complex functions as the primary energy source and is indispensable for many physiological functions including nerve conduction [7], muscle contraction [8], and blood pressure regulation [9]. 

Exercise regulates Mg distribution and utilization while Mg participates in strength activities and cardiorespiratory functions [1,10,11,12], indicating the reciprocal relation between exercise and Mg in the human body [11]. In response to exercise, Mg is being transported to locations where energy production is taking place [10]. For example, during long-term endurance exercise, serum Mg is likely to shift from serum to erythrocytes or muscle to support exercise function [1]. On the other hand, short-term exercise may reduce plasma/serum volume, resulting in the elevation of serum Mg levels [1].

The role of Mg in muscle function has been studied extensively [9,10,11,13,14]. Mg participates in the process of energy metabolism and assists the maintenance of normal muscle contraction and relaxation. Muscle performance is positively associated with serum Mg levels in elderly [15] and male athletes [16,17]. In addition, studies have shown that Mg deficiency may lead to the distortion of neuromuscular function [18,19,20], suggesting a possible association between Mg and muscle cramps. Although not seen in other populations, the benefit of Mg supplementation in reversing cramps has been observed in pregnant women [14]. However, evidence is lacking in relation to exercise-associated muscle cramps [14]. 

Mg is rich in certain types of foods including nuts, seeds, fruits, vegetables and whole grains (Table 1). The Recommended Dietary Allowance (RDA) for Mg is 400–420 mg for males and 310–320 mg for females above 19 years old [21]. Despite Mg’s critical role in energy metabolism, maintaining Mg intake at an adequate level has been frequently overlooked among the general population and athletes [11,22]. The demand for Mg is likely to increase during accelerated metabolic situations, thus, physically active individuals may have higher Mg requirements in order to maintain optimal exercise performance as compared with their inactive peers [11,13]. For individuals who participate in a strength training program, a suboptimal or even deficient Mg status may lead to inefficient energy metabolism and decreased endurance [23]. In aerobic exercise, higher intake of Mg has been shown to be associated with less oxygen needs and better cardiorespiratory indices [24,25].

Since the improvement of exercise performance has been observed in individuals with Mg supplementation [1,10,18,26,27,28], it is of great importance to raise the awareness of Mg intake in physically active populations. Of note, Mg supplementation has been widely recommended to physically active individuals, especially athletes. However, the effectiveness of such practice on exercise performance is less elucidated. Although a large body of studies have examined the relation between Mg and physical performance [1,9,11,13,22,23], the outcome measurement varies, which primarily includes physiological indicators such as lactate levels, blood pressure, heart rate and VO_2_ max [25,29]. The purpose of this review is to evaluate the association between Mg status/supplementation and exercise performance with direct measures such as grip strength and knee torque.

## 2. The Association between Mg and Exercise Performance

### 2.1. Evidence from Animal Studies

Exercise can cause the shift of energy substrates in the body [30]. For example, moderate exercise may decrease the glucose concentration while increasing the levels of lactate [31], which results in decreased efficiency of working muscle [32]. A number of studies have been conducted in animal models (e.g., rats and gerbils) to investigate the effects of Mg supplementation on the change of glucose and lactate in brain, blood, and muscle in response to exercise [18,26,27,32,33,34] (Table 2). All animals followed the protocols of forced exercise that mimicked the physical training in humans [27]. One forced swimming study showed that gerbils with Mg sulfate (90 mg/kg, ip) 30 min before exercise experienced a rapid increase of glucose concentrations and delayed accumulation of lactate in the brain compared to that in the control group [32]. Since the brain regulates the downstream physiological function and the metabolic activities in the brain increased during exercise and [35], the enhanced glucose utilization due to Mg supplementation may result in improved exercise performance [32]. In addition, three studies examined peripheral glucose levels in exercising gerbils or rats [18,27,33]. The results showed a dramatic boost of the plasma glucose level in the Mg treatment group, which resulted in elevated glucose availability during exercise. Moreover, Mg supplementation was demonstrated to attenuate the accumulation of lactate in blood, and therefore delay muscle fatigue [18,27,33]. Furthermore, there was a significant extension of the swimming time among gerbils/rats in the Mg treatment group as compared with those animals in the control group. Notably, Mg supplementation was shown to maintain plasma glucose at a relatively high level post exercise, suggesting that Mg may assist in muscle tissue recovery [27].

A recent study systematically investigated the change of glucose level in blood, muscle, and the brain in treadmill exercising rats and found that Mg improved exercise performance via increasing glucose availability and decreasing the accumulation of lactate [26]. Of note, the results indicated a spike of muscle glucose levels in the Mg treatment group [26]. As compared to baseline, the glucose level in muscle increased to 650–780% during exercise [26]. In addition, the muscle lactate increase was delayed in the Mg treatment group during exercise while the production rate of lactate in the brain was significantly increased—approximately two-fold as much as that in the control group [26]. However, there was no significant difference in blood lactate levels between the Mg treatment group and the control group [26].

In sum, animal studies suggest that Mg supplementation can improve exercise performance via regulating the concentration of glucose and lactate in the brain, muscle, and in circulation [18,26,27,32,33,34]. Higher availability of Mg will increase glucose levels in both the central nervous and peripheral systems during exercise [18,26,27,32].

### 2.2. Evidence from Human Studies

To date, data directly linking Mg status to exercise performance in humans are sparse. Human studies have linked serum/plasma Mg levels or Mg supplementation to various exercise-related outcomes including bench press, hand grip, maximal isometric trunk flexion, heart rate, and energy needs. Physiological indices have been used as the proxy measures of exercise performance [28,29,36,37,38,39]. For example, in one randomized controlled trial [40], the researchers randomly divided 30 healthy individuals (aged 18–22 years) into three groups: (1) Mg supplementation group; (2) Mg supplementation + Tae-Kwan-Do training group; and (3) Tae-Kwan-Do training group. After a 4-week intervention, the results indicated that Mg supplementation improved exercise performance, measured by a 20 m shuttle run test, through decreasing the accumulation of lactate, which was consistent with the findings from animal studies [18,26,27,32,33].

One direct indicator of exercise performance is muscle strength. A few cross-sectional surveys [15,16,17] and randomized control trials (RCTs) [23,36,41] reported a positive association of serum Mg levels/Mg supplementation with muscle strength, but other studies indicated no significant effect [42,43] (Table 3). A cross-sectional analysis using data from the InCHIANTI (aging in the Chianti area) study [15] found that serum Mg concentrations were positively associated with grip strength, lower-leg power, knee extension torque, and ankle extension strength in the elderly after adjustment for potential confounders such as age, body mass index and physical activity levels [15]. Also, one cross-sectional survey conducted in male athletes indicated a positive association of Mg intake with maximal isometric trunk flexion, rotation, hand grip, jumping performance, and all isokinetic strength indicators [16]. Another survey study has demonstrated the change in intercellular water (ICW) was associated with strength reduction in athletes and this association could be modified by Mg levels, indicating that Mg may improve muscle strength [17]. 

RCTs are considered to be the best approach to establish a causal relationship. A few RCTs were conducted to examine the association between Mg supplementation and exercise performance [23,41,42,43]. However, findings were inconsistent. In a 7-week strength training intervention, participants were randomly assigned to either receive Mg supplementation or a placebo. Post-assessments indicated that quadriceps strength was improved in both groups, while the strength gain in the Mg supplement group was greater than that in the control group (*p* value < 0.05) [23]. Specifically, the quadriceps torque increased from 2.38 ± 0.80 to 3.07 ± 0.92 in the Mg treated group as compared to 2.35 ± 0.43 to 2.58 ± 0.43 (newton meter (Nm)/kg) in the control group [23]. Another study was conducted in elderly women who participated in a 12-week exercise program [43]. At the end of study, Mg supplementation significantly improved the participants’ gait speed (Mg: ∆ = 0.21 ± 0.27 m/s vs. control: 0.14 ± 0.003 m/s, *p* value = 0.0006) and chair stand times (Mg: ∆ = −1.21 ± 1.86 s vs. control: −1.31 ± 0.33 s, *p* value < 0.0001). It is noted that the increased walking speed may have great implications for exercise benefits as it is an important indicator for diagnosing degenerative lean muscle loss [43]. However, no difference was observed for isometric knee extension torque or handgrip strength between the experimental group and the control group [43]. In addition, two interventional studies investigated whether Mg supplementation might affect physiological and exercise indicators with both aerobic and resistance exercises [28,36]. After 14 days of Mg supplementation, a larger reduction in BP was observed in the post-exercise group (8.9 mm Hg for resting BP and 13 mm Hg for post-exercise BP) [28]. Moreover, studies demonstrated that short-term (one week) Mg supplementation (350 mg/day) may be sufficient to improve exercise performance [33].

However, still another two RCTs failed to observe any beneficial effects of Mg supplementation on exercise performance [41,42]. In one study among middle-aged overweight women, no significant difference in the changes for handgrip and knee extension were found between the Mg supplementation group and the control group after 8-week follow-up [41]. For example, while the individuals in the control group increased their handgrip from 26.7 ± 5.0 to 27.8 ± 4.7 kg, participants from the Mg treatment group increased their strength from 26.3 ± 5.5 to 27.8 ± 4.5 kg [41]. The other study investigated the effects of Mg supplementation in marathon runners [42]. During the 10-week study period (4 weeks before the marathon and 6 weeks after), Mg supplementation failed to provide any benefits to supplement users’ marathon performance. Also, the researchers didn’t observe any significant difference in Mg concentration between the two groups measured either in blood or muscle [42]. Therefore, they concluded that little physiological or exercise improvement was likely, due to the unsuccessful alteration of serum Mg concentrations by supplementation [42]. 

## 3. Possible Mechanism

During exercise, carbohydrates, fats, and protein are broken down sequentially to provide energy and support muscle movement. The underlying mechanism of Mg-induced strength improvement is possibly due to the function of Mg in protein synthesis and energy metabolism [11], contributing to the process of muscle contraction and relaxation [36]. Additional Mg supply may decrease the oxygen requirements for muscle cells during exercise, leading to optimization of physical movements [44]. 

Glucose is the primary source of energy, and the need for glucose will increase during exercise [10,13,45]. In general, glycogen is broken down rapidly in muscles to support physical movement and, once muscle glycogen is depleted, blood will transport glucose from the liver or kidney to the muscle for continuous energy supply. Glucose is converted to pyruvate during aerobic exercise, and is reduced to lactate during anaerobic exercise [32]. The accumulation of lactate may induce muscle fatigue, and thus affect exercise performance [18]. 

Glucose metabolism and glycolysis are key processes for energy production during exercise [30]. According to animal studies, Mg may influence exercise performance via the glucose metabolic pathway [18,26,27,32]. Energy production depends upon cellular Mg status, since Mg-ATP is the direct molecule used in all physical activities. The process is impaired when there is insufficient Mg supply. In general, Mg plays an important role in glucose metabolism through (1) glucose homeostasis pathways; (2) regulating phosphorylation; and (3) acting as the cofactor for many key enzymes such as pyruvate dehydrogenase and creatine kinase [46]. Studies have indicated that a low Mg diet is associated with impaired glucose metabolism and Mg consumption is inversely related to the risk of type 2 diabetes [46,47]. In a study examining the effect of magnesium supplementation on glucose and insulin levels in sportsmen at rest or at exhaustion [48], the researchers found that 4-week Mg supplementation was beneficial to glucose utilization at both sedentary and active phases [48]. 

The brain requires more glucose as the energy source during exercise in coordinating all functional movements and managing physiological fluctuations [26]; meanwhile, exercise induces the flux of Mg from the brain to plasma. Since Mg plays a key role in regulating cerebral glucose levels and downstream neuronal activity, decreased Mg levels may lead to the decline of exercise performance as a result of glucose depletion [32]. In animal models, Mg supplementation increases glucose availability in the muscle, blood, and brain, and reduces lactate accumulation in blood and muscle, but not in the brain [26]. Because lactate is a metabolite of glucose, it is hypothesized that lactate may act as an alternative fuel in the brain in response to the increased energy metabolism during exercise [26]. Thus, lactate may function as the fuel for neurons when glucose is insufficient [49]. Additionally, when glycogen is depleted, lactate may regulate the gluconeogenesis pathways by facilitating the utilization of gluconeogenic substrates [50].

## 4. Potential Confounders and Methodological Issues

Several intervention studies in humans failed to detect any effects of Mg in strength training programs. However, this does not necessarily mean Mg supplementation has no impact on exercise performance. First, it has been suggested in the literature that the fluctuation of blood Mg levels is closely related to different types of exercise [13]. While short-term exercise typically increases Mg concentration in the blood, long-term strenuous exercise may reduce blood Mg levels [13], because intense physical exercise such as marathons and triathlons can cause greater loss of Mg from all body compartments (erythrocyte, blood, etc.) [11]. Together, these may at least in part explain the null effect that researchers observed among marathon runners with Mg supplementation [42]. Second, it is possible that the dosage of the supplement was not potent in inducing any detectible improvement of exercise performance. For example, no improvements in the muscle function in a group of marathon runners injected daily with 126 mg elemental Mg [42] and no significant gains in muscle strength with daily 250 mg Mg supplementation in middle-aged overweight women were observed [41]. It is speculated that the selected dose might not be sufficient [41,42].

Of note, the impact of individuals’ background Mg levels on exercise performance is unclear. Some research suggests that exercise performance may improve only if an individual’s baseline Mg levels is low [11]. However, one study tested this hypothesis and found that baseline serum Mg concentrations was not a critical factor in determining exercise performance in response to Mg supplementation [41]. Another study excluded individuals with hypomagnesemia (serum Mg concentrations below 1.8 mg/dL) and still found a significant association between serum Mg levels and muscle strength [15]. Therefore, future research in physically active individuals should also include pre-assessment of Mg status, and consider baseline Mg levels while investigating the association between Mg supplementation and exercise performance.

In addition, several other issues need to be considered. First, the selected Mg supplementation and dosages are inconsistent across different human studies. While three RCTs used Mg oxide, two others used Mg citrate and Mg-L-aspartate hydrochloride, respectively. The corresponding dosage of element Mg ranged from 122.6 mg/day to 8 mg/kg body weight/day. Thus, lacking a standard dosage for Mg supplementation might partially explain the inconsistent findings from human studies. Second, all RCTs were conducted in special populations with a small sample size. Except for one study in the elderly, which included over 100 participants, most studies had a sample size ranging from 13 to 69. Third, most studies that utilized a training program included only two groups: exercise only and exercise plus Mg. A 2 × 2 factorial design including four groups: (1) a group with neither exercise nor Mg supplementation; (2) an exercise only group; (3) an Mg supplementation only group; and (4) a group with both, is recommended, so that the main effects of exercise and Mg supplementation as well as their interaction can be examined. With only two groups, as seen in the previous studies, one cannot rule out the possibility that the training program competes against Mg supplementation’s effect on the muscle strength, and therefore the estimate of Mg effect may be biased.

## 5. Summary and Future Perspectives

In summary, Mg is an essential mineral involved in energy metabolism, cardiorespiratory function, and muscle actions. The general population, even physically active individuals, is documented to have insufficient Mg intake. Exercise performance may be compromised with deficient Mg levels. Findings from animal studies suggested Mg supplementation may improve the efficiency of energy metabolism, while human studies indicated Mg supplementation may improve performance parameters in both aerobic and anaerobic exercises. However, more rigorous future studies, especially large-scale intervention studies in humans, are needed to establish the causal relationship. 

## Figures and Tables

**Table 1:** Major dietary sources of magnesium^1^.

Food Item	Amount for One Serving	Magnesium, mg/Serving
Almonds, dry roasted	1 oz	80
Spinach, boiled	½ cup	78
Cashews, dry roasted	1 oz	74
Soy milk, plain or vanilla	1 cup	61
Black beans, cooked	½ cup	60
Edamame, shelled, cooked	½ cup	50
Peanut butter, smooth	2 tbsp	49
Bread, whole wheat	2 slices	46
Avocado, cubed	1 cup	44
Potato, baked with skin	3.5 oz	43
Rice, brown, cooked	½ cup	42
Yogurt, plain, low fat	8 oz	42
Oatmeal, instant	1 packet	36
Kidney beans, canned	½ cup	35
Banana	1 medium	32
Salmon, Atlantic, farmed, cooked	3 oz	26
Milk	1 cup	24–27
Chicken breast, roasted	3 oz	22
Beef, ground, 90% lean, pan boiled	3 oz	20
Broccoli, chopped and cooked	½ cup	12
Apple	1 medium	9

^1^ Source: https://ods.od.nih.gov/factsheets/Magnesium-HealthProfessional/. Oz, ounce; tsbp, tablespoon.

**Table 2:** Summary of animal studies.

Source	Subject	Treatment Group	Control Group	Main Results
Cheng et al. [32], 2007	Gerbils	90 mg kg^−1^ Mg Sulfate, intraperitoneal injection	90 mg kg^−1^ Saline solution, intraperitoneal injection	↑ cerebral glucose and pyruvate ↓ cerebral lactate formation
Chen et al. [27], 2009	Sprague-Dawley Rats	90 mg kg^−1^ Mg Sulfate, intraperitoneal injection	90 mg kg^−1^ Saline solution, intraperitoneal injection	↓ retention frequencies in treadmill exercise (only in the high-speed group) ↑ higher plasma glucose post exercise ↓ lower plasma lactate post exercise
Cheng et al. [18], 2010	Gerbils	90 mg kg^−1^ Mg Sulfate, intraperitoneal injection	90 mg kg^−1^ Saline solution, intraperitoneal injection	↑ duration of swimming ↑ plasma magnesium ↑ plasma glucose ↓ plasma lactate formation
Chen et al. [33], 2010	Gerbils	90 mg kg^−1^ Mg Sulfate, intraperitoneal injection	90 mg kg^−1^ Saline solution, intraperitoneal injection	↑ plasma glucose ↓ plasma lactate formation
Wang et al. [34], 2014	Gerbils	Nigari 18 mg·kg^−1^, orally	double-distilled water	↓ retention frequencies in treadmill exercise
Chen et al. [26], 2014	Sprague-Dawley Rats	90 mg kg^−1^ Mg Sulfate, intraperitoneal injection	90 mg kg^−1^ Saline solution, intraperitoneal injection	↑ glucose in blood, muscle and brain ↓ lactate formation in blood and muscle ↑ lactate formation in brain

**Table 3:** Summary of human studies.

Source	Study Design	Group	Treatment	No. of Participants	Age, year	Male, %	Main Findings
Santos et al. [16] (2011, Portugal)	Cross-sectional, seven-day diet record	Male athletes	NA	26	20.1 ± 4.9	100	Positive association between Mg intake and strength performance
Matias et al. [17] (2010, Portugal)	Cross-sectional, one month	Male athletes	NA	20	22.9 ± 2.9	100	Mg supplementation can attenuate the strength reduction due to decreased ICW
Dominguez et al. [15] (2006, Italy)	Cross-sectional analysis of the baseline data from a prospective cohort study	Elderly	NA	1138	66.7 ± 15.2	46	Serum Mg level is positively associated with muscle performance in elderly
Kass and Poeira [36] (2015, UK)	Randomized, double-blind, cross-over, placebo controlled	T1	300 mg/day for 1 week (acute)	6	35.8 ± 6.2	50	Short-term supplementation was associated with better exercise performance
		T2	300 mg/day for 4 weeks (chronic)	7	40.8 ± 4.4	57	
Veronese et al. [41] (2014, Italy)	RCT	T	300 mg/day for 12 weeks	53	71.8 ± 5.0	0	Daily magnesium oxide supplementation improves physical performance in healthy elderly women
		C	Blank control without treatment	71	71.3 ± 5.4	0	
Moslehi et al. [43] (2013, Iran)	RCT	T	250 mg/day for 8 weeks	35	46.5 ± 3.8	0	Supplementation has no significant impact on muscle strength gain
		C	Placebo	34	46.1 ± 4.6	0	
Ternlanche et al. [42] (1992, South Africa)	RCT	T	122.6 mg/day for 10 weeks	10	32.4 ± 11.5	NA	Supplementation did not improve exercise performance. It also did not improve muscle recovery
		C	Placebo	10	32.5 ± 7.7	NA	
Brilla and Haley [23] (1992, USA)	RCT	T	Mg intake 8 mg/kg body weight per day	12	NA	NA	Supplementation led to greater quadriceps torque
		C	Placebo	14	NA	NA	

^1^ C, control; ICW, intercellular water; NA, not applicable/available; RCT, randomized controlled trial; T, treatment.

## References

[1] Bohl C.H., Volpe S.L. (2002). Magnesium and exercise. Crit. Rev. Food Sci. Nutr..

[2] George G.A., Heaton F.W. (1978). Effect of magnesium deficiency on energy metabolism and protein synthesis by liver. Int. J. Biochem..

[3] Littlefield N.A., Hass B.S., McGarrity L.J., Morris S.M. (1991). Effect of magnesium on the growth and cell cycle of transformed and non-transformed epithelial rat liver cells in vitro. Cell Biol. Toxicol..

[4] Garfinkel L., Garfinkel D. (1985). Magnesium regulation of the glycolytic pathway and the enzymes involved. Magnesium.

[5] Dorup I., Clausen T. (1991). Effects of magnesium and zinc deficiencies on growth and protein synthesis in skeletal muscle and the heart. Br. J. Nutr..

[6] Mildvan A.S. (1987). Role of magnesium and other divalent cations in ATP-utilizing enzymes. Magnesium.

[7] Mert T., Gunes Y., Guven M., Gunay I., Ozcengiz D. (2003). Effects of calcium and magnesium on peripheral nerve conduction. Pol. J. Pharmacol..

[8] Potter J.D., Robertson S.P., Johnson J.D. (1981). Magnesium and the regulation of muscle contraction. Fed. Proc..

[9] Newhouse I.J., Finstad E.W. (2000). The effects of magnesium supplementation on exercise performance. Clin. J. Sport Med..

[10] Lukaski H.C. (2001). Magnesium, zinc, and chromium nutrition and athletic performance. Can. J. Appl. Physiol..

[11] Nielsen F.H., Lukaski H.C. (2006). Update on the relationship between magnesium and exercise. Magnes. Res..

[12] Laires M.J., Monteiro C.P., Matias C.N., Santos D.A., Silva A.M., Bicho M. (2014). Magnesium status and exercise performance in athletes. Trace Elem. Electroly.

[13] Rayssiguier Y., Guezennec C.Y., Durlach J. (1990). New experimental and clinical data on the relationship between magnesium and sport. Magnes. Res..

[14] Garrison S.R., Allan G.M., Sekhon R.K., Musini V.M., Khan K.M. (2012). Magnesium for skeletal muscle cramps. Cochrane Database Syst. Rev..

[15] Dominguez L.J., Barbagallo M., Lauretani F., Bandinelli S., Bos A., Corsi A.M., Simonsick E.M., Ferrucci L. (2006). Magnesium and muscle performance in older persons: the InCHIANTI study. Am. J. Clin. Nutr..

[16] Santos D.A., Matias C.N., Monteiro C.P., Silva A.M., Rocha P.M., Minderico C.S., Bettencourt Sardinha L., Laires M.J. (2011). Magnesium intake is associated with strength performance in elite basketball, handball and volleyball players. Magnes. Res..

[17] Matias C.N., Santos D.A., Monteiro C.P., Silva A.M., Raposo Mde F., Martins F., Sardinha L.B., Bicho M., Laires M.J. (2010). Magnesium and strength in elite judo athletes according to intracellular water changes. Magnes. Res..

[18] Cheng S.M., Yang L.L., Chen S.H., Hsu M.H., Chen I.J., Cheng F.C. (2010). Magnesium sulfate enhances exercise performance and manipulates dynamic changes in peripheral glucose utilization. Eur. J. Appl. Physiol..

[19] Keen C.L., Lowney P., Gershwin M.E., Hurley L.S., Stern J.S. (1987). Dietary magnesium intake influences exercise capacity and hematologic parameters in rats. Metabolism.

[20] Liu L., Borowski G., Rose L.I. (1983). Hypomagnesemia in a Tennis Player. Phys. Sportsmed..

[21] Magnesium-Fact Sheet for Health Professionals. https://ods.od.nih.gov/factsheets/Magnesium-HealthProfessional/.

[22] Volpe S.L. (2015). Magnesium and the Athlete. Curr. Sport Med. Rep..

[23] Brilla L.R., Haley T.F. (1992). Effect of Magnesium Supplementation on Strength Training in Humans. J. Am. Coll. Nutr..

[24] Lukaski H.C. (2004). Vitamin and mineral status: effects on physical performance. Nutrition.

[25] Finstad E.W., Newhouse I.J., Lukaski H.C., McAuliffe J.E., Stewart C.R. (2001). The effects of magnesium supplementation on exercise performance. Med. Sci. Sports Exerc..

[26] Chen H.Y., Cheng F.C., Pan H.C., Hsu J.C., Wang M.F. (2014). Magnesium Enhances Exercise Performance via Increasing Glucose Availability in the Blood, Muscle, and Brain during Exercise. PLoS ONE.

[27] Chen Y.J., Chen H.Y., Wang M.F., Hsu M.H., Liang W.M., Cheng F.C. (2009). Effects of magnesium on exercise performance and plasma glucose and lactate concentrations in rats using a novel blood-sampling technique. Appl. Physiol. Nutr. Metab..

[28] Kass L.S., Skinner P., Poeira F. (2013). A pilot study on the effects of magnesium supplementation with high and low habitual dietary magnesium intake on resting and recovery from aerobic and resistance exercise and systolic blood pressure. J. Sports Sci. Med..

[29] Setaro L., Santos-Silva P.R., Nakano E.Y., Sales C.H., Nunes N., Greve J.M., Colli C. (2014). Magnesium status and the physical performance of volleyball players: effects of magnesium supplementation. J. Sport Sci..

[30] Coyle E.F. (1995). Substrate utilization during exercise in active people. Am. J. Clin. Nutr..

[31] Miller B.F., Fattor J.A., Jacobs K.A., Horning M.A., Navazio F., Lindinger M.I., Brooks G.A. (2002). Lactate and glucose interactions during rest and exercise in men: effect of exogenous lactate infusion. J. Physiol..

[32] Cheng S.M., Yang D.Y., Lee C.P., Pan H.C., Lin M.T., Chen S.H., Cheng F.C. (2007). Effects of magnesium sulfate on dynamic changes of brain glucose and its metabolites during a short-term forced swimming in gerbils. Eur. J. Appl. Physiol..

[33] Chen I.J., Cheng S.M. (2010). Effects of magnesium sulfate on dynamic changes in blood glucose levels and glucose transporter-3 expression in the Striatum during short-term forced swimming in Gerbils. Int. J. Sport Exerc. Sci..

[34] Wang M.L., Chen Y.J., Cheng F.C. (2014). Nigari (deep seawater concentrate) enhances the treadmill exercise performance of gerbils. Biol. Sport.

[35] Ahlborg G., Wahren J. (1972). Brain substrate utilization during prolonged exercise. Scand. J. Clin. Lab. Investig..

[36] Kass L.S., Poeira F. (2015). The effect of acute vs chronic magnesium supplementation on exercise and recovery on resistance exercise, blood pressure and total peripheral resistance on normotensive adults. J. Int. Soc. Sports Nutr..

[37] Peveler W.W., Palmer T.G. (2012). Effect of magnesium lactate dihydrate and calcium lactate monohydrate on 20-km cycling time trial performance. J. Strength Cond. Res..

[38] Cinar V., Nizamlioglu M., Mogulkoc R., Baltaci A.K. (2007). Effects of magnesium supplementation on blood parameters of athletes at rest and after exercise. Biol. Trace Elem. Res..

[39] Lukaski H.C., Nielsen F.H. (2002). Dietary magnesium depletion affects metabolic responses during submaximal exercise in postmenopausal women. J. Nutr..

[40] Cinar V., Nizamlioglu M., Mogulkoc R. (2006). The effect of magnesium supplementation on lactate levels of sportsmen and sedanter. Acta Physiol. Hung..

[41] Veronese N., Berton L., Carraro S., Bolzetta F., De Rui M., Perissinotto E., Toffanello E.D., Bano G., Pizzato S., Miotto F. (2014). Effect of oral magnesium supplementation on physical performance in healthy elderly women involved in a weekly exercise program: a randomized controlled trial. Am. J. Clin. Nutr..

[42] Terblanche S., Noakes T.D., Dennis S.C., Marais D., Eckert M. (1992). Failure of magnesium supplementation to influence marathon running performance or recovery in magnesium-replete subjects. Int. J. Sport Nutr..

[43] Moslehi N., Vafa M., Sarrafzadeh J., Rahimi-Foroushani A. (2013). Does magnesium supplementation improve body composition and muscle strength in middle-aged overweight women? A double-blind, placebo-controlled, randomized clinical trial. Biol. Trace Elem. Res..

[44] Golf S.W., Bender S., Gruttner J. (1998). On the significance of magnesium in extreme physical stress. Cardiovasc Drugs Ther.

[45] Kjaer M. (1998). Hepatic glucose production during exercise. Adv. Exp. Med. Biol..

[46] Barbagallo M., Dominguez L.J. (2015). Magnesium and type 2 diabetes. World J. Diabetes.

[47] Song Y., Manson J.E., Buring J.E., Liu S. (2004). Dietary magnesium intake in relation to plasma insulin levels and risk of type 2 diabetes in women. Diabetes Care.

[48] Cinar V., Polat Y., Mogulkoc R., Nizamlioglu M., Baltaci A.K. (2008). The effect of magnesium supplementation on glucose and insulin levels of tae-kwan-do sportsmen and sedentary subjects. Pak. J. Pharm. Sci..

[49] Choi I.Y., Seaquist E.R., Gruetter R. (2003). Effect of hypoglycemia on brain glycogen metabolism in vivo. J. Neurosci. Res..

[50] Brooks G.A. (2002). Lactate shuttles in nature. Biochem. Soc. Trans..

